# From the archives: the shape of flowers, timely flowering, and floral organ longevity

**DOI:** 10.1093/plcell/koaf028

**Published:** 2025-02-03

**Authors:** Regina Mencia

**Affiliations:** Assistant Features Editor, The Plant Cell, American Society of Plant Biologists; Instituto de Agrobiotecnología del Litoral (CONICET-UNL), Cátedra de Biología Celular y Molecular, Facultad de Bioquímica y Ciencias Biológicas, Universidad Nacional del Litoral, Santa Fe 3000, Argentina

## 2000: AGL15 and floral organ longevity

Floral organ identity arises from the specific expression of floral genes in the floral meristem and whorls of developing flowers, according to the well-known (and often modified) ABC model of flower development (Bowman and Moyroud 2024). Many floral organ identity genes belong to the MADS-box family of transcription factors. AGAMOUS-like 15 (AGL15) in the MADS domain family is highly expressed in the early embryo in *Brassica napus* and therefore was thought to play a key role mainly in developing embryos. However, Fernández et al. (2000) found that AGL15 accumulates at the shoot apex and the base of leaf petioles during the vegetative phase and later accumulates in floral buds during the reproductive phase. They showed that constitutive or ectopic expression of *AGL15* led to prolonged longevity of sepals and petals and delayed the transition to flowering, floral organ abscission, and fruit maturation, suggesting an important role in floral organ development and longevity. It was later found that AGL15 and AGL18 act together to repress the floral transition in Arabidopsis (*A. thaliana*) (Adamczyk et al. 2007) by forming a complex that activates the expression of the microRNA miR156 (Serivichyaswat et al. 2015). Kim (2014) identified other MADS-box transcription factors involved in floral organ abscission, highlighting their remarkable versatility and broad regulatory functions in plant development, particularly floral development.

## 2020: The Nup96-HOS1 complex and timely flowering

Plants must perceive environmental cues such as light and temperature to accurately time the transition to flowering and ensure successful reproduction. A key molecular regulator of flowering time is the transcription factor CONSTANS (CO). CO accumulates in leaves, where it directly activates the expression of *FLOWERING LOCUS T* (*FT*), which travels to the shoot apical meristem to initiate floral primordia differentiation. CO activity is tightly controlled at multiple levels, including transcriptional regulation, protein-protein interactions, and protein stability. A complex network of positive and negative regulators modulates *CO* transcription, and CO activity is influenced by interactions with both activating and inhibitory partners. In addition, CO stability is regulated through proteasome-dependent degradation (Takagi et al. 2023). Under long-day photoperiods, the E3 ubiquitin ligase HIGH EXPRESSION OF OSMOTICALLY RESPONSIVE GENE1 (HOS1) mediates CO degradation in the morning, thereby repressing flowering. Cheng et al. (2020) discovered that HOS1 interacts with the nuclear pore complex through Nucleoporin96 (Nup96). Loss of Nup96 function destabilizes HOS1, leading to early flowering phenotypes in *nup96*, *hos1*, and the *nup96 hos1* double mutant. The early flowering in these mutants is largely attributed to CO overaccumulation. These findings revealed a mechanism in which the Nup96-HOS1 complex mutually stabilizes its components, thereby limiting CO levels and preventing premature flowering under long-day conditions.

## 2024: The role of *PhDFE* in shaping flowers

Petals are essential floral structures with diverse and intricate shapes and colors, playing important ecological roles, particularly in attracting and interacting with specific pollinators. In *Petunia x hybrida*, mature petals are fused, forming a corolla divided into 2 domains: the tube and the limb. *PhDEFICIENS* (*PhDEF*) is expressed in the floral meristem epidermal and mesophyll cell layers. However, the layer-specific contribution of PhDEF remained unclear until a recent study by Chopy et al. (2024). They investigated how restricting *PhDEF* expression to specific layers affects petal shape. The authors examined 2 types of *Petunia x hybrida* mutants: *wico*, with *PhDEF* expression limited to the epidermis, and *star*, with expression restricted to inner mesophyll cells. The mutants displayed strikingly different morphologies: *wico* flowers developed a significantly reduced tube but a nearly normal limb, whereas *star* flowers formed a normal tube but a highly reduced limb (see Figure). These findings suggest that petal morphogenesis in *Petunia x hybrida* is modular and depends on layer-specific *PhDEF* expression. The study also revealed that *PhDEF* directly binds to the *ANTHOCYANIN2* (*AN2*) terminator, a major regulator of anthocyanin biosynthesis, indicating a role in regulating petal pigmentation. This work lays a foundation for further exploration of the genetic mechanisms underlying complex petal development.

**Figure.**
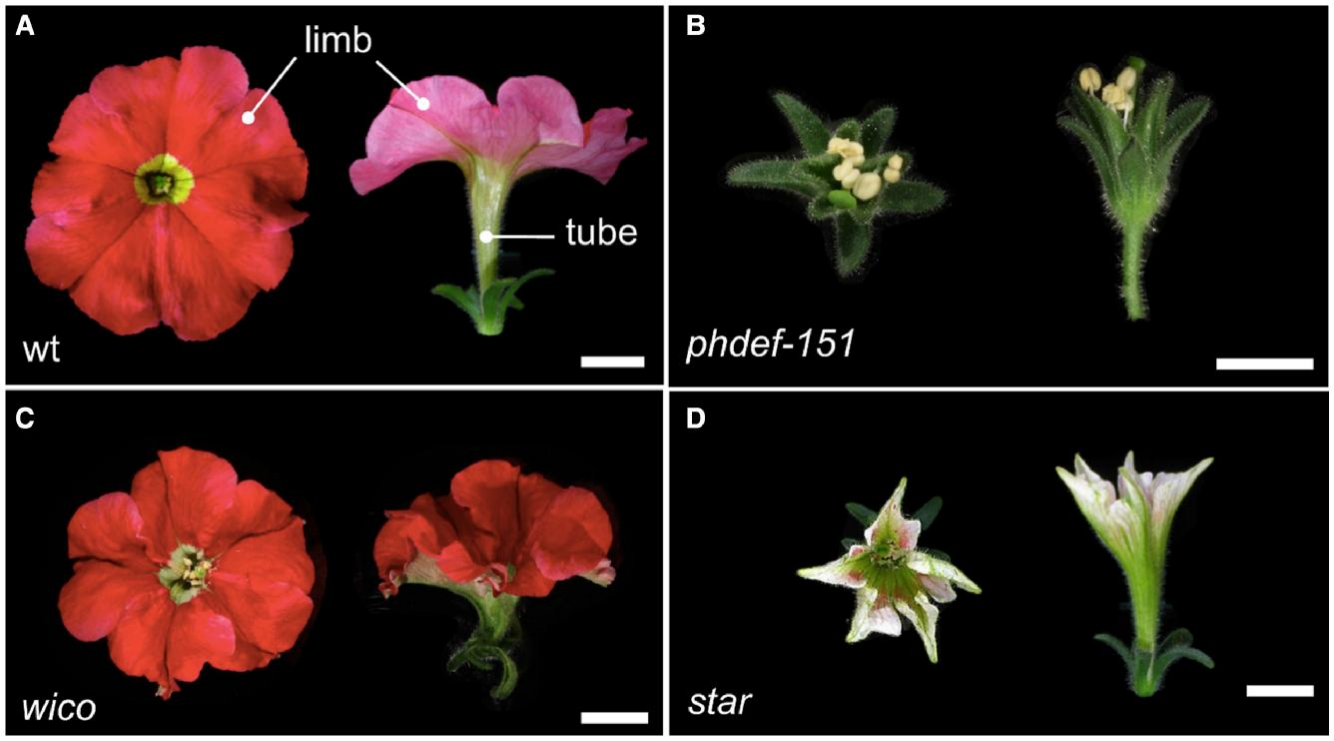
Representative *Petunia x hybrida* flowers phenotypes: Wild-type **(A)**, *phdef-151*  **(B)** display a complete homeotic conversion of petals into sepals, *wico*  **(C)** display significantly reduced tube but a nearly normal limb, and *star*  **(D)** display normal tube but a highly reduced limb. Scale bar: 1 cm. Adapted from Chopy et al. (2024), Figure 1.

## Dive Curated Terms

The following phenotypic, genotypic, and functional terms are of significance to the work described in this paper:

AGL15 Gramene: AT5G13790
AGL15 Araport: AT5G13790

AGL18 Gramene: AT3G57390
AGL18 Araport: AT3G57390

floral organ identity Planteome: TO:0006019

HOS1 Gramene: AT2G39810
HOS1 Araport: AT2G39810
